# A measure of success

**DOI:** 10.1186/2045-3701-2-35

**Published:** 2012-10-16

**Authors:** Kuan-Teh Jeang

**Affiliations:** 1 The National Institutes of Health, Bethesda, MD, USA

## Abstract

*Cell and Bioscience* is on track to receive its first Impact Factor in mid-2013. What is the role of the Impact Factor as a measure of a journal’s success?

## 

A thought provoking commentary was published recently that proposes the use of several metrics to predict the future H-index of a scientist and accordingly the likelihood of his/her “success” [1]. Using a dataset that encompasses 3,085 neuroscientists, 57 Drosophila researchers, and 151 evolutionary biologists, the authors collated and analyzed history of publication, citations and funding. Based on inputs that include current H-index, the number of published articles, the years since first publication, the number of published papers in “prestigious” journals, and the number of distinct journals in which the papers are published, the authors constructed an algorithm ( http://klab.smpp.northwestern.edu/h-index.html) with which they claim reasonable success in predicting a researcher’s future H-index. This methodology is an intriguing endeavor. Time will tell whether it has the same value as, or better value than, some of the stock charting programs used by Wall Street investment advisors to direct investment dollars into various equities.

For journal editors, there is unfortunately no innovative proposal to predict a journal’s future success. In 2011, the Society of Chinese Bioscientists in America (SCBA) launched *Cell and Bioscience *[2,3] as the flagship journal of the Society. The intent was to establish a high quality, highly visible, Open Access journal for the Society and for the at-large international bioscience community. One reason there was a need for *Cell and Bioscience* is the rapid rise of bioscience and biomedical research in Asia [4]. Indeed, a 2011 Royal Society Report predicted that by 2013, the global share of total scientific articles published from China will outpace articles from the United States [5]. *Cell and Bioscience* is aptly positioned at the nexus of burgeoning scientific interactions between Asia and the rest of the world.

As of this writing, *Cell and Bioscience* has been publishing for nearly two calendar years. The journal has been tracked and indexed by PubMed ( http://www.Pubmed.gov), Scopus ( http://www.scopus.com), and was recently added for tracking by ISI Thomson-Reuters to its Web of Science, science citation index. What this means is that every article in *Cell and Bioscience* since its launch in January 2011 has now been tracked by Web of Science, science citation index and that the journal will receive its first Journal Citation Report, Impact Factor in mid-2013. The Impact Factor is a measure of the citation frequency of the articles published in a journal and is often used as a proxy to reflect the standing of a journal in its field. There are certain misconceptions about what an Impact Factor number means or does not mean [6]. However, it should be noted that garnering an Impact Factor is a first step for a new journal to be measured against its established peers. In this respect, the editors and editorial board of *Cell and Bioscience* intend to compete vigorously and successfully in this and other measures of journal excellence.
